# Prevalence and Genotype of *Trichomonas vaginalis* among Men in Xinxiang City, Henan Province, China

**DOI:** 10.1155/2023/4119956

**Published:** 2023-02-28

**Authors:** Zhenchao Zhang, Yuhui Sang, Pucheng Wu, Yujia Shang, Lesong Li, Yujuan Duan, Linfei Zhao, Minghui Gao, Lihua Guo, Xiaowei Tian, Zhenke Yang, Shuai Wang, Lixia Hao, Xuefang Mei

**Affiliations:** ^1^Xinxiang Key Laboratory of Pathogenic Biology, School of Basic Medical Sciences, Xinxiang Medical University, Xinxiang, Henan 453003, China; ^2^Xinxiang Maternity and Child Healthcare Hospital, Xinxiang, Henan 453003, China

## Abstract

*Trichomonas vaginalis* (*T. vaginalis*) could cause trichomoniasis through sexual transmission, which was globally distributed. In this study, the prevalence and phylogenetic analyses of *T. vaginalis* among men in Xinxiang were conducted. From October 2018 to December 2019, a total of 634 male clinical samples were collected, including 254 samples of semen, 43 samples of prostate fluid, and 337 samples of urine. These samples were examined by nested PCR and a total of 32 (5.05%) *T. vaginalis*-positive samples were detected. Among these samples, the positive rates of *T. vaginalis* in semen, prostate fluid, and urine were 7.87% (20/254), 4.65% (2/43), and 2.97% (10/337), respectively. Three actin genes were successfully isolated and sequenced from the 32 positive DNA samples, and the analysis of the sequence and phylogenetic tree showed that the three actin gene sequences exhibited 99.7%–100% homology to the published actin gene sequence (EU076580) in NCBI, and the *T. vaginalis* strains in the three positive samples were identified as genotype E. Our results demonstrate a notable genotype of *T. vaginalis* in the male population and provide insight into the performance of these genetic markers in the molecular epidemiology of trichomoniasis. However, further studies are needed to research the association between the genotype and the pathogenicity of *T. vaginalis*.

## 1. Introduction

*T. vaginalis* is a flagellated protozoan that parasitizes the urogenital tract of humans, which causes nonviral sexually transmitted disease. The World Health Organization (WHO) estimated that there were about 156 million cases of trichomoniasis worldwide in 2016, accounting for almost half of the incidence of globally transmitted infections that year [1]. *T. vaginalis* parasitizes the vagina and urethra of women, causing *Trichomonas* vaginitis, cervicitis, and urethritis, increasing the risk of pregnancy complications, preterm delivery, and cervical cancer [2–4], and can also lead to infertility [5, 6]. Moreover, *T. vaginalis* is also parasitic on the male urethra and prostate gland, causing urethritis, prostatitis, and epididymitis [5]. The infection of *T. vaginalis* increases the risk of male infertility and prostate cancer [7–9]. In addition, trichomoniasis can also increase the risk of HIV transmission by two or three times [5, 10, 11]. However, 85% of women and 77% of men with *T. vaginalis* are asymptomatic [12].

Despite the public health importance and global distribution of trichomoniasis, there are still many unanswered questions about the various features of the *T. vaginalis* infection. Understanding the genetic characteristics of *T. vaginalis* is valuable for the prevention and control of human trichomoniasis [13]. Currently, a number of methods have been developed to study gene polymorphism of *T. vaginalis* isolates, which include multilocus sequence typing (MLST), microsatellite (MS) genotyping, and internal transcribed spacer (ITS) typing [14, 15]. Phylogenies have already witnessed applications in numerous practical domains, such as in epidemiology [16], multiple sequence alignment [17], and prediction of gene and protein function [18, 19]. Homology analysis of genomic sequences can be used to construct phylogenetic trees to evaluate the genetic diversity and evolutionary relationships of *T. vaginalis* [20, 21]. High levels of sequence similarity can consistently produce accurate evolutionary trees [22].

So far, there are few studies on the genetic diversity and molecular epidemiology of *T. vaginalis* in China, and the infection data of male trichomoniasis has been lacking. In order to understand the infection status and genetic diversity of *T. vaginalis* in Xinxiang City, Henan Province, China, we evaluated the prevalence of *T. vaginalis* in men by detecting the trophozoites of *T. vaginalis* in male semen, prostate fluid, and urine with nested PCR. The products amplified by nested PCR were sequenced to analyze the genotype of *T. vaginalis* prevalent in men.

## 2. Materials and Methods

### 2.1. Ethics Statement

The study was reviewed and approved by the Ethics Review Committee of Xinxiang Medical University (Reference No. XYLL-2018S006).

### 2.2. Sample Collection

From October 2018 to December 2019, we collected 634 samples, including 254 semen, 43 prostatic fluid, and 337 urine samples. The prostatic fluid, urine, and semen were collected from men who went to the hospital outpatient clinic for routine examination.

### 2.3. DNA Extraction

The samples of semen and prostatic fluid were centrifuged at 2000 rpm for 5 min. The collected urine was allowed to stand at room temperature for 1 hour, discarded the supernatant, and then centrifuged at 2000 rpm for 5 min. The precipitation was used to extract DNA with Universal Genomic DNA Kit (CWBIO, Beijing, China) according to the manufacturer's instructions. The DNA sample was preserved by freezing at −20°C before analyses.

### 2.4. Nested PCR

The actin gene (GenBank: AF237734) was selected as the target gene of nested PCR [23–25]. According to the primer sequences reported in the literature [24, 26], as shown in Table 1, the external primers (Tv8S and Tv9R) and internal primers (Tv10S and Tv11R) were synthesized by Wuhan GeneCreate Biological Engineering Co., Ltd. The length of the amplified target gene fragment was 1100 bp. PCR amplification was performed in two steps by a thermocycler (Bio-Rad, USA). The first stage of the PCR master mixture consisted of 1 *μ*l DNA template, 12.5 *μ*l 2 × Taq Plus Master Mix (Vazyme, Nanjing, China), 2 *μ*l external primers (20 pmol each of Tv8S and Tv9R), and distilled water to adjust the final volume to in 25 *μ*l. The second stage of the PCR master mixture was composed of 2 *μ*l first stage PCR products, 25 *μ*l 2 × Taq Plus Master Mix, 4 *μ*l internal primers (20 pmol each of Tv10S and Tv11R), and distilled water in 50 *μ*l of the final volume. The first amplification procedure consists of 35 cycles, and each cycle is composed of denaturation (95°C for 15 s), annealing (55°C for 15 s), and extension (72°C for 1 min). The denaturation at 95°C was conducted for 3 min before the first cycle, and the final extension was conducted at 72°C for 5 min after the last cycle. There were also 35 cycles in the second step. The denaturation and extension are the same as the first step, but the annealing temperature is 50°C. After PCR, we analyzed 10 *μ*l PCR product through electrophoresis on 1% agarose gel in Tris-borate-EDTA buffer (TBE and pH 8.3) and then visualized it under UV light with GoldView I Nuclear Staining Dyes (Solarbio, Beijing, China).

The length of the target gene was 1100 bp, which is only 28 bp shorter than the size of the actin gene's open reading frame. The PCR products of positive samples were separated by 1% agarose gel electrophoresis, and the target DNAs were recovered by E.Z.N.A.® Gel Extraction Kit (OMEGA, USA).

### 2.5. Sequence Analysis

All recovered DNA products were sequenced by Sanger technology (GeneCreate, Wuhan, China) to determine the base sequence of the target gene. Clustal W was used to edit and align the sequence (https://www.ebi.ac.uk/Tools/msa/clustalo/), which was compared with the reference sequences (EU076580, AF237734, EU076582, EU076584, EU076579, EU076578, EU076585, EU076583, EU076586, and AB468096) [23] obtained from GenBank by basic local alignment search tool (BLAST). To analyze the genotype of *T. vaginalis*, we constructed a phylogenetic tree with MEGA software (version 7.0) [27] using the neighbor-joining (NJ) algorithm, containing sequences representing *T. vaginalis* isolates in GenBank.

### 2.6. Statistical Analysis

Differences in *T. vaginalis* prevalence for different variables were analyzed using a chi-square test. Statistical analysis was performed using SPSS 20 software for Windows (SPSS Inc., Chicago, Illinois, USA). The differences were considered statistically significant if *p* < 0.05.

## 3. Results

### 3.1. Nested PCR

**Figure 1:**
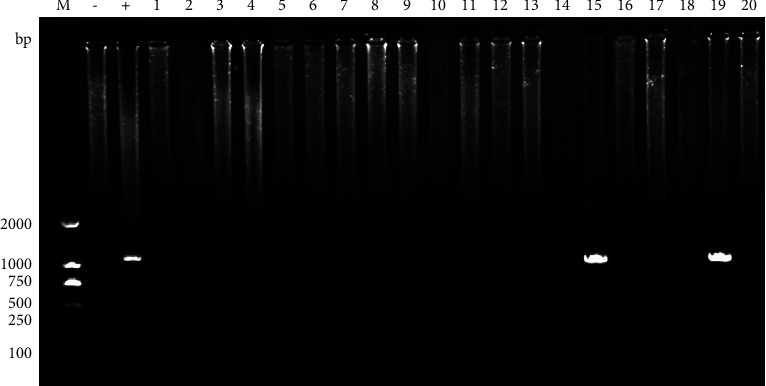
Gel electrophoresis analysis of nested PCR amplification products of *T. vaginalis* actin gene. M 2000 bp DNA marker; −: negative control; +: positive control; lane 1–20: the amplification products of *T. vaginalis* actin gene from clinical samples.

The actin gene of *T. vaginalis* was amplified by nested PCR, and agarose gel electrophoresis indicated that 1100 bp fragment was visualized among the *T. vaginalis* positive sample. There was no difference in the length of the positive amplicons (Figure 1). All the positive DNA samples were recovered for sequencing.

### 3.2. Prevalence of *T. vaginalis* in Men

As shown in Table 2, a total of 634 samples were tested in this present study, and the total positive rate of *T. vaginalis* was 5.05%. Moreover, the positive rates were different in the male genitourinary tract samples of semen (7.87%), prostatic fluid (4.65%), and urine (2.97%), respectively. This result indicated that the positive rate of *T. vaginalis* in semen was significantly higher than that in prostatic fluid and urine (*p* < 0.05).

### 3.3. Sequence and Phylogenetic Analyses

**Figure 2:**
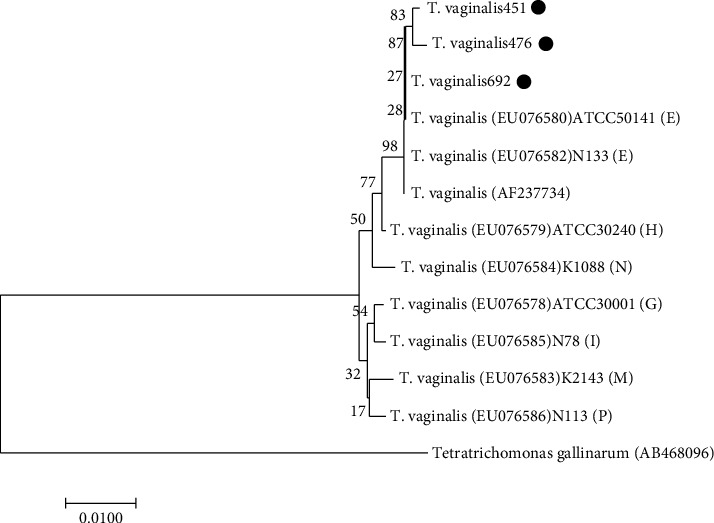
A phylogenetic tree of Xinxiang and reference trichomonad isolates was built by the neighbor-joining method using mega 7.0, based on the actin gene. The three sequences identified in this study are shown with a dark dot.

To evaluate the genetic diversity among *T. vaginalis* isolates from men, we sequenced the 32 positive DNA samples, but only 3 sample DNAs were sequenced successfully, which were submitted to GenBank (Accession Number: MZ014497, MZ014498, and MZ014499). We conducted a multiple alignments of the various *T. vaginalis* actin genotypes with the sequences found in this study and reference isolates and constructed a tree with the NJ algorithm (Figure 2). Sequence analysis indicated that the percentages of the similarity between the three sequences and the reference sequence in GenBank (Accession Number: EU076580) were 99.7%, 99.8%, and 100%, respectively, which showed high sequence homology. According to the genetic distance between sequences, we draw the branch length in proportion. To assess the reliability of this tree, bootstrap analysis was carried out with 1000 replicates. Phylogenetic diversity analysis showed that the 451, 476, and 692 strains of *T. vaginalis* were sequenced successfully in the study with a dark dot belonged to an independent branch, and the three strains belong to genotype E.

## 4. Discussion

In 2016, WHO estimated that the global infection rate of trichomoniasis was 0.6% in males and 5.3% in females [1]. It has been reported that more than 276.4 million people worldwide have been infected with *T. vaginalis* [28, 29]. In 2013, the prevalence of trichomoniasis was estimated to be about 58 million cases, and women were twice as likely to be infected as men [7]. The latest epidemiological data on the prevalence of trichomoniasis in women and men in the United States were released in 2018 [30]. The results showed that the infection rate among American adults (ages 18–59) from 2013 to 2014 was 0.5% for males and 1.8% for females. On the other hand, there are significant ethnic differences in *T. vaginalis* between African-American men and women, with an estimated prevalence of 6.8% in the black population and 0.4% in the rest of the population. Seike et al. [31] found that the infection rate of *T. vaginalis* was 1.4% in Japanese male patients with urethritis and 1.0% in men without urethritis. Seo et al. [32] found that the infection rate of *T. vaginalis* was 4% in Korean men, 2.4%–8.2% in Croatian men with urethritis, and 1% in asymptomatic men with trichomoniasis [23]. In Iran, various studies have determined that the prevalence of trichomoniasis is between 2% and 8% and can reach more than 30% depending on culture and social status [25].

The results of this study showed that the infection rate of *T. vaginalis* was 5.05% in males, which is higher than that of the United States, Japan, and South Korea, lower than that of African Americans, and similar to that of Iran. Patel et al. conducted an epidemiological study on *T. vaginalis* infection in the United States, indicating that the number of sexual partners was positively correlated with the rate of male *T. vaginalis* infection [30]. In addition, some studies showed that the prevalence of *T. vaginalis* was correlated with seasons to some extent, and the prevalence rate of *T. vaginalis* in the autumn and winter was higher than that in spring and summer [23, 33]. Samples collected in this study were also concentrated in autumn and winter. Besides, patients with low immunity could also lead to a high rate of *T. vaginalis* infection. It is impossible for us to determine the number of sexual partners of these men and the time of infection, so a long-term investigation is needed to understand the overall natural history of *Trichomonas* infection.

In an epidemiological study on *T. vaginalis* infection in the United States, the rate of *T. vaginalis* infection in males was lower than that in females [30]. Not so in Xinxiang city, the infection rate of male was higher than that of female. Due to the limited number of samples collected in this experiment, the data may be limited to some extent. Furthermore, we failed to obtain the subjects' age, occupation, ethnicity, educational background, sexual partner status, and whether there are other sexually transmitted diseases and failed to discuss from relevant aspects, which is also one of the limitations of this experiment.

In this study, we found that *T. vaginalis* infection was more common among men in Xinxiang, and our results showed that the positive detection rate of *T. vaginalis* was different in different parts of the urogenital tract. The positive rate of the semen sample was the highest, while the positive rate of the urine sample was the lowest. Moreover, in a global epidemiological study of *T. vaginalis*, a zinc-rich environment in the prostate may prevent *T. vaginalis* from continuing infection, and the detection rate of *T. vaginalis* should be low [34]. The low positive detection rate of urine samples may be due to the dilution of *T. vaginalis* by a large amount of urine. It may also be that the patient has discharged urine before collecting urine samples, resulting in the loss of *T. vaginalis* in subsequent urine sample collection. In addition, because the subjects might suffer from other urogenital diseases and the parasitic position of *T. vaginalis* might change with the time of infection, the type of positive specimens may not fully reflect the anatomical site of infection [35]. Therefore, the precise location of *T. vaginalis* in the male genitourinary tract is worth further study to determine a sample type with a high detection rate.

Molecular typing can provide information about the genetic diversity, population structure, and epidemiological links of *T. vaginalis* in the population [36, 37]. In addition, the investigation of genetic diversity helps to identify the source of pathogenicity, drug resistance, and recurrent infection of the parasite [24, 38, 39]. PCR-RFLP based on the actin gene has been extensively studied for the genotyping of *T. vaginalis* strains. In Turkey, the majority of *T. vaginalis* isolates were the actin genotype E (45%), the remaining isolates were genotype G (7.5%), genotype N (1.5%), and genotype H (1.5%), two of which were mixed genotypes E and H (10%) [40]. In Kenya, five actin genotypes were revealed by RFLP analysis, and the researchers used the latter method to detect five genotypes: E (50%), G (13.6%), I (4.5%), N (27.3%), and P (4.5%) [41]. However, the overall prevalence of trichomoniasis in northern Iran was low. The major genotypes were E (0.2%), G (0.2%), and I (0.08%) [42]. Based on the sequence of the 18S rRNA gene, the genetic variation of *T. vaginalis* was found in Anyang, Zhengzhou, Shangqiu, Luoyang, Pingdingshan, Zhumadian, and Xinyang of Henan Province. The results indicated that *T. vaginalis* isolated from central China could be considered as a single population [43]. Genotype E (58.82%), H (17.65%), mixed 1 (17.65%), and mixed 2 (5.88%) were identified as the *T. vaginalis* genotypes of the female infected in Xinxiang [23]. In this study, 3 out of 32 positive DNA samples were sequenced successfully, and these sequences were used to analyze genotypes. The low success rate of sequencing may be caused by the low content of positive DNA. In our previous study [23], *T. vaginalis* was isolated and cultured, and then the trophozoites were harvested and extracted DNA. Phylogenetic analysis showed that *T. vaginalis* 451, 476, and 692 belonged to genotype E and existed in the same clade. This indicates that the infection of *T. vaginalis* between males and females in Xinxiang City has a high correlation. According to sequence alignment and phylogenetic analysis, the level of gene diversity was observed. We observed the nucleotide changes between the genotype E sequence and the reference genome. Sequence alignment showed that the “C” nucleotide was replaced by the “G” nucleotide (C ⟶ G) in the nucleotide sequence site 356 of *T. vaginalis* 451 and the site 355 of *T. vaginalis* 476. The substitution of nucleotide sequence site 774 of *T. vaginalis* 451 and the site 171 of *T. vaginalis* 476 were A ⟶ G. In addition, the site 290 of *T. vaginalis* 476 changed from T to G.

Some studies have shown that the genotype of *T. vaginalis* originally comes from two different evolutionary branches [15, 44, 45]. However, only genotype E was found in our study, and no other actin genotypes of *T. vaginalis* (A, G, H, I, M, N, and P) were found. Due to the limitations of research methods, we could not obtain the gene sequences of all positive samples. In addition, the sample size was limited. Therefore, it is necessary to further study whether there are other genotypes of *T. vaginalis* in male infection in Xinxiang. More studies are needed in the future to clarify the association between certain genotypes and clinical manifestations. Previous research has established that metronidazole resistance is related to the mutation of the actin gene [45]. Bradic et al. found that two metronidazole-resistant single nucleotide changes occurred in the actin-like gene of *Trichomonas foetus* [46]. Therefore, regular genotyping of *Trichomonas* in different areas to master the latest information on the relationship between *Trichomonas* genotypes and drug resistance will help us better understand the drug resistance process of *T. vaginalis*, which is conducive to the prevention and control of trichomoniasis.

## 5. Conclusion

This is the first report on the prevalence and molecular characterization of *T. vaginalis* in men from Xinxiang. The results showed that the infection of *T. vaginalis* was common in men in Xinxiang. Phylogeny showed that the prevalent genotypes of *T. vaginalis* in men in the Xinxiang area were mainly type E, and all of them existed in the same evolutionary branch. These findings provide insights for evaluating the performance of genetic markers in the molecular epidemiology of trichomoniasis. Further research is needed to improve our understanding of the epidemiology of *T. vaginalis* and to explore the relationship between genotypes and clinical manifestations.

## Figures and Tables

**Table 1:** Oligonucleotide primer sequences used for nested PCR in this research.

Name	Sequences (5′⟶3′)	Description
Tv8S	TCTGGAATGGCTGAAGAAGACG	Forward primer for the actin gene of *T. vaginalis* in the first stage
Tv9R	CAGGGTACATCGTATTGGTC	Reverse primer for the actin gene of *T. vaginalis* in the first stage
Tv10S	CAGACACTCGTTATCG	Forward primer for the actin gene of *T. vaginalis* in the second stage
Tv11R	CGGTGAACGATGGATG	Reverse primer for the actin gene of *T. vaginalis* in the second stage

**Table 2:** The detection rate of *T. vaginalis* in different types of samples in Xinxiang.

Sample type	No. of positive	Sample size	Prevalence (%)	95% CI	*p* values
Semen	20	254	7.87	4.5–11.2	0.026
Urine	10	337	2.97	1.2–4.8	
Prostatic fluid	2	43	4.65	−1.9–11.2	
Total	32	634	5.05		

## Data Availability

The data supporting the conclusions of this article are included within the article. The sequences generated in the present study were submitted to the GenBank database under the accession numbers MZ014497, MZ014498, and MZ014499.
